# Dr. Walker, on Vision

**Published:** 1809-10

**Authors:** John Walker

**Affiliations:** Salisbury Court


### *To the Editors of the Medical and Physical Journal.*

GENTLEMEN,

I Wish to offer to the notice of your Readers, a statement on the subject of Vision, which I have occasionally made to some eminent physiologists and anatomists in this Metropolis. Without doubting the reality of the facts which I stated to them, they all professed them to have escaped their observation. Perhaps, some of your readers may have observed them, and may be able to account for them. Of the facts I am perfectly assured, and now go to state them; after which, I may venture a conjecture on their cause.

If, after an unduly extended vigil; if after more than usual exercise; if after more than a moderate use of wine, all of which may occur in the case of travelling all night; I look at such objects along the road in the morning, as hedges, woods, &c. a thousand fantastic shapes, of the most undefined and evanescent figure, seem to hover before my enfevered view; as I remember to have, once, happened to me in approaching Chantilly, from Paris. If, at such time, I look at any ordinary range of railing or pallisadoes; or, at a gate formed of a succession of vertical bars, the vision is not much disturbed; but, if I cast my eyes on a similar arrangement of parallel lines, in a horizontal direction, they are dazzled beyond all endurance.

These different effects of parallel lines, on the enfevered eye, accordingly as they are presented to it, in a vertical or or horizontal direction, are, most distinctly noticeable in viewing large capital engravings, like those of Rooker or Lowry. One may look, with some composure, on the columns of architecture, &c. where the tincts are made out with vertical lines; but the masses of sky or water, produced by horizontal lines, it is impossible to gaze upon with stedfastness. A very fine artist, (of whom I was a pupil) in drawing, etching, and engraving figures and landscape, pointed out to me, in ascertaining which of the masses of lines, crossing each other in an engraving, were produced by the *aqua fortis*; that it was only necessary to hold the print, in such direction, that the etched lines should become horizontal. I have often tried the experiment, and found that those parallel lines which were brought to bear on the eye, in this way, became a great deal more distinct than the others.

A few days ago, in reading a proof, there occurred a word in it (Talleyrand) in which I thought there were two ls. The printing was very small; the light but faint, at the time; I could not be certain. I bethought me of the engraver's method of obtaining most distinct retinal impression in the undisturbed eye. I turned the proof so, that the lines of it fell vertically on my eye. The lls were, consequently, transverse to the line of my nose. I at once saw there were two, and put upon one of them my *deleatur*.

The facts are before you. How are they to be accounted for? At this I only conjecture. What is the object or final cause? Of this I have no idea. On this most wonderful part of the animal economy, I seem to be calling your attention to the very threshold of the temple of intelligence.

Externally, the optician can explain to us the porch, its partitions, their forms, how occupied, &c. He has constructed instruments on their principles. The physiologist can give us some idea of the changes in this wonderful porch, necessary for the admittance of the different messengers, as they come from distances more or less remote; or, as they are more or less vividly clothed with light; or rather, as they are, literally, rays more or less refrangible, more or less, I will say, intensely mixed, or vivid.

Internally, we are at once lost, if we attempt to comprehend the hidden economy.

The observation may appear trite, and is applicable, no doubt, to the simplest laws of physiology. If attraction, tion, the first law of creation, if crystallization, whatever modification of it this may be; if these, in the inanimate creation, in unorganized nature, be incomprehensible, the simplest laws of physiology must be ever beyond our reach. But I allude not here, to the general doctrine of the impressions on the retina, passing to the sensorium, through the nerves; nor, to the clumsy doctrine of there being any necessity for decussation of nerve, for rectifying what has been supposed to be an *error loci aut positionis*, in objects being inverted on the retina; I allude to the more tangible peculiarities of the optic nerve bearing the messages to the sensorium. At the time of writing this, the characters arise on my view. I turn my head to the right; then back again; then to the left. The letters appear just the same in these different positions. I close one eye, right or left, and observe the same effects. Now this same optic nerve, this grand road between the visible and intellectual world, between the optical porch and the most secret recesses of mind, must have been in very different conditions of tension, on turning my head, as already described.

On turning it, e. g. to the left, must not the optic nerve of the left eye become stretched? yet I find not its vision to become more acute. Must not the optic nerve of the right eye become relaxed? yet I find not its vision to become less acute.

When, at any time, we bring our eyes to bear, most earnestly, on any particular object; when we bring our utmost attention to it, we seem, at that particular instant, only capable of distinctly seeing one particular point in that object, (it is almost a mathematical point, a nothing at all.) A small portion of line from such point, to the right and left, will make more sensible impression on the retina, than the same portion in a perpendicularly transverse direction to it from the same point. This is the doctrine; now, for the conjectures.

Is not the retina an expansion of the optic nerve, fibres going off from it radially, at the place of its entering into the formation of the eye? Are not these indefinitely ramified as they spread over the great chamber of the vitreous humour, where they may be infinitely reticulated with each other, and with the every where present cellular tissue, or universally connecting membrane? Will not a line presented to the eye, as an object of vision, be more sensibly felt by the optic nerve, or will it not excite or stimulate it more, if its image on the retina fall, fall, coincidently, on any of the radial fibres of the optic nerve, than if it fell upon them transversely? If so, then let us consider what would be the effect, on our vision, of a different disposition, in the organs of it.

If the optic nerve entered the eye immediately opposite to the pupil, we should not, there, possess any distinct point of direct vision; an object, directly before it, would be invisible. Each eye would be as unguarded against the direct approach of an extraneous body, as is that of a one-eyed man against the oblique approach of an object, coming downwards and inwards upon the pupil; in which way, I think, I have read of a one-eyed man losing his sight from a thorn, in passing a quickset hedge. In viewing, directly, any right line, it would fall (the centre of it excepted, which would be invisible) on, directly, the radial fibre, on both sides of the darkened spot; producing, probably, a painful degree of excitement. We could not then, as at present, fixing our attention on a small field of vision before us, let the great picture of the hemisphere, duly painted on the retina, lie unheeded. Every object, on whatever part of the retinæ, would, along the corresponding radial fibres of each, rush to the optic nerves and sensorium. Objects, the most remote from the centre of the hemisphere before us, would thus be incessantly obtrusive upon the attention.

If the optic nerve entered the eye at any distance from the point of direet vision, in a vertical direction, upwards or downwards, a line falling on the pupil, in a vertical direction, would produce the greatest excitement, and give the strongest vision; if it entered the eye at any distance from the direct point, in a lateral direction, inwards or outwards, a line in a horizontal direction, would, as at present, give the strongest vision of such object. In both instances it would, probably, produce a dazzling effect.

As the eyes are now constructed, a right line cannot be presented to them, the image of which shall cross the darkened spots of each eye, and directly or coincidentally fall on the nervous fibres radiating from them. The line that would cross the darkened spot of one eye would be remote from it in the other.

The darkened spots are more nearly horizontal than vertical to the points of direct vision. The radial fibres, consequently, pass through the points in a direction more approaching the horizontal than the vertical direction. To this I attribute the more distinct vision of horizontal than of vertical lines. Perhaps, men with only one eye have most most distinct vision of right lines, when they bear upon that eye in such oblique direction, as to fall on the point of direct vision coincidentally with the radial fibres passsing through it from the place of insertion of the optic nerve. If the fact, pointed out to me by the ingenious artist in Dublin, have been hitherto unnoticed by physiologists in general, the conjecture may be not irrational; I therefore venture to offer it. Perhaps some of your readers, of leisure, may have the opportunity of determining this not absolutely incurious point in the physiology of the eye.

---

Your Journal being professedly physical as well as medical, physiological, &c. I now go to offer you some observations on a sort of little optical illusion, which every body must have observed, and which may have often been accounted for; but as I do not remember to have ever heard or seen any explanation of the little phænomenon, I still retain the simple ideas which I first conceived of the cause when a youth. I think, on passing parallel railing at a distance, where all seems fixed and regular, if any object happens to intervene, suppose a post or bough of a tree, that part of the railing which we see directly beyond the sides of the intervening object becomes distorted or irregular. The same appearances take place on a smaller scale, if in viewing the parallel lines in a print, we pass a pen, pencil, pin, or any other body, from line to line between the eye and the print. I shew my want of reading, perhaps, in not knowing how this is explained. I once entertained the notion, and I still suppose it may be a correct one, that the atmosphere is condensed on the surface of all hard bodies, by the more direct, or rather firm, resistance which they make to its pressure, than the body of elastic air, occupying their place on their removal can do. If this notion be correct, then, in looking at parallel lines, as already described, or at any other object, we see them as through a lens.

JOHN WALKER.

*Salisbury Court, 23, viii, 1809.*

